# Association Between the 25-Hydroxyvitamin D Status and Physical Performance in Healthy Recreational Athletes

**DOI:** 10.3390/ijerph15122724

**Published:** 2018-12-03

**Authors:** Cornelia Zeitler, Robert Fritz, Gerhard Smekal, Cem Ekmekcioglu

**Affiliations:** 1 Department of Environmental Health, Center for Public Health, Medical University of Vienna, Kinderspitalgasse 15, 1090 Vienna, Austria; c.zeitler@hotmail.com; 2 Sportordination, Alser Straße 27/1/6, 1080 Vienna, Austria; fritz@sportordination.com; 3 Institute of Sport Sciences, University of Vienna, Auf der Schmelz 6a, 1150 Vienna, Austria; gerhard.smekal@univie.ac.at

**Keywords:** vitamin D, maximal performance, submaximal performance, 25-hydroxyvitamin D (25(OH)D), physical activity, treadmill ergometer, athlete

## Abstract

Molecular and clinical studies have linked vitamin D (vitD) deficiency to several aspects of muscle performance. For this retrospective cross-sectional study data from 297 male (M) and 284 female (F) healthy recreational athletes were used to evaluate the prevalence of vitD deficiency in athletes living in Austria and to determine whether serum 25-hydroxyvitamin D (25(OH)D) correlates with maximal (P_max_) and submaximal physical performance (P_submax_) measured on a treadmill ergometer. The data were controlled for age, season, weekly training hours (WTH), body mass index (BMI) and smoking status. 96 M and 75 F had 25(OH)D levels ≤ 20 ng/mL. 25(OH)D levels showed seasonal variations, but no seasonal differences in P_max_ and P_submax_ were detected. M with 25(OH)D levels ≤ 20 ng/mL had significantly lower P_submax_ (*p* = 0.045) than those with normal levels. In F no significant differences in P_max_ or P_submax_ were detected. Stepwise multiple regression analysis including all covariates revealed significant correlations between 25(OH)D levels and P_max_ (β = 0.138, *p* = 0.003) and P_submax_ (β = 0.152, *p* = 0.002) in M. Interestingly, for F significant correlations between 25(OH)D and both P_max_ and P_submax_ disappeared after adding WTH to the model. In conclusion, our data suggest that 25(OH)D status is associated with physical performance especially in M, while in F, WTH and BMI seem to affect the correlation.

## 1. Introduction

The discovery of vitamin D and its role in musculoskeletal pathways at the end of the 20th century has led to a significant decline in cases of rickets in children, with the awareness about vitamin D deficiency and its consequences getting a second boost at the moment [1].

Vitamin D is known for its critical role in musculoskeletal health through maintaining a homeostasis of calcium and phosphate by enhancement of their absorption in the small intestine [2] thus preventing bone fractures and falls, especially in older populations [3,4]. Moreover it has been proposed to play an important part in reducing the risk of multiple types of cancer [5,6] as well as several autoimmune diseases [7,8] and osteoarthritis [9]. A recent meta-analysis of observational studies by Ekmekcioglu et al. [6] showed that higher 25(OH)D levels are associated with lower risks for type 2 diabetes mellitus and colorectal cancer. Additionally vitamin D deficiency was shown to be associated with higher blood pressure [10], obesity [11] and higher blood lipid levels [12].

Out of the many forms in which vitamin D presents itself, vitamin D_2_ (ergocalciferol) and vitamin D_3_ (cholecalciferol) are the physiologically most important ones. While vitamin D_3_ can be found in fatty fish, egg yolks, liver and dairy products, mushrooms are a source for vitamin D_2_ [13]. The main source for vitamin D however, is the synthesis by the skin in reaction with UVB light. The metabolic steps in the liver and kidney leading to formation of the main circulating metabolite 25-hydroxyvitamin D (25(OH)D) and the active metabolite 1,25-dihydroxyvitamin D (1,25(OH)_2_D) are regulated through multiple feedback mechanisms and are still under investigation [14].

According to recent findings the enzymatic systems needed to perform the activating hydroxylation step as well as the nuclear hormone receptor, vitamin D receptor (VDR), were detected in several cells all over the body, including muscle tissue [15]. Subsequently it is assumed that 1,25(OH)_2_D also influences skeletal muscle via molecular pathways [16].This hypothesis was tested in numerous observational and interventional studies. Most of the studies have been conducted in elderly populations, linking vitamin D deficiency to several aspects of muscle strength and performance such as handgrip, lower limb strength, balance, timed up and go test and gait speed [17,18,19]. However, also divergent results have been reported, showing no improvements in strength parameters following vitamin D supplementation in elderly populations [20,21].

Especially in the last two decades there have been increased efforts to determine the role of vitamin D in physical performance in younger populations and athletes. Vitamin D levels have been associated with various aspects of muscle strength and recovery in these populations as well, including for example hand-grip strength, gastro-soleus strength and walking distance in a group of young vitamin D deficient Asian Indians [22], hand-grip strength in a group of severely vitamin D deficient Somali women [23], isometric strength and relative grip strength in young hockey players [24], vertical jump and reduced injury risk after winter vitamin D supplementation in elite ballet dancers [25], 10 m sprint times and vertical jump height following supplementation in a sample of vitamin D deficient young athletes [26] as well as improved phosphocreatine recovery half-time of the soleus muscle in severely deficient individuals [27] and enhanced recovery following intense exercise in active male adults [28]. Again, several studies have yielded contradictory results showing no association between vitamin D levels and muscle strength parameters [29,30,31,32].

In accordance with the rising interest on vitamin D, study groups all over the world have conducted research to determine the prevalence of vitamin D deficiency, with alarming results. According to Hilger et al. [33] 88.1% of examined samples worldwide showed mean 25(OH)D levels beneath 75 nmol/L (30 ng/mL; conversion factor: 1 ng/mL = 2.496 nmol/L), 37.3% with mean levels <50 nmol/L (20 ng/mL) and 6.7% with mean levels <25 nmol/L (10 ng/mL). Similar prevalence rates have been found for athletes [34]. However, a huge variability in all the collected data partly limits the significance of possible estimates on vitamin D levels.

Considering that there has not been a universal consensus on optimal vitamin levels yet, the comparison of studies investigating the results of insufficient vitamin D status on different health outcomes is particularly challenging. While the US Institute of Medicine Committee (IOM) proposes that 20 ng/mL (50 nmol/L) as a cut-off level meets the needs of almost all of the general population [35], the US Endocrinology Society regards any 25(OH)D levels under 30 ng/mL (75 nmol/L), and above 20 ng/mL (50 nmol/L) as insufficient [36].

The aim of this trial was to evaluate the prevalence of vitamin D deficiency in a large sample representing healthy recreational athletes in Austria and to determine whether vitamin D status correlates with both, maximal and submaximal physical performance on a treadmill ergometer.

## 2. Materials and Methods

The study was approved by the ethics committee of the Medical University of Vienna (EK-Number 1929/2016). The data used for this study were generated in “Sportordination”, a multidisciplinary medical centre for sports medicine and sports sciences, located in 1080 Vienna, Austria. On their first visit all athletes had to fill out a questionnaire on their medical history, weekly training hours and personal best results at races. Following the medical check-up height, body weight and body fat were measured. Afterwards athletes performed an incremental performance test on a treadmill ergometer to subjective exhaustion supervised by a sports scientist. To ensure valid test results, all athletes were asked to refrain from doing any sports, drink enough fluid and have a full carbohydrate-based supper on the day prior to the performance test and to make sure that their last food intake was two hours before the test. According to the gathered information on training habits and race performance, the fitting protocol for the performance test was chosen. For athletes with half marathon minimum times under 2 h or 10 km minimum times under 60 min the following protocol was used: Initial running speed started at 6 km/h with increments of 2 km/h every 3 min. This protocol has been proven sufficient for incremental performance tests of amateur athletes [37,38].

At the beginning of the test blood lactate and glucose concentration were determined from capillary blood drawn from the earlobe at rest using “SUPER GL ambulance” (Dr. Müller Gerätebau GmbH, Freital, Germany), a glucose and lactate measurement unit working with a compact sensor. During the test, the treadmill was stopped after each exercise level to determine lactate and glucose concentrations. Heart rate was measured continuously using telemetered electrocardiogram (ECG) recordings and blood pressure was checked regularly. P_max_ was defined as highest possible speed (km/h) until subjective exhaustion. While maximal performance represents the overall fitness, submaximal performance is far more important to athletes as it determines their training status. During an incremental performance test lactate levels form a characteristic curve [39,40] with a slight increase at the beginning due to accumulating lactate as long as heart rate and capillary dilatation have not adapted, and then stabilization just above baseline when oxidative phosphorylation is responsible for the mean part of energy production. There is a rise in lactate levels as the intensity increases and anaerobic glycolysis begins to take part in ATP production. Within this transition between aerobic and anaerobic energy production oxidative energy supply is no longer sufficient to keep up with the rising need for ATP. The capacity of mitochondria is pushed to its limits resulting in exponential rise in lactate levels as pyruvate production exceeds lactate clearance marking the individual anaerobic threshold (IAT). By measuring glucose levels in addition to lactate only we were able to be more precise in continuously determining which energy substrate was used. We calculated IAT using the concept of Dickhut et al. [41], one of several lactate threshold concepts that can be used to determine submaximal performance and has been proven sufficient for performance diagnostics [42,43].

All healthy recreational athletes aged 18 to 65, who had performed their performance diagnostic test on a treadmill ergometer between 1 July 2013 and 1 February 2017 with valid data on serum 25(OH)D levels were included in the study. 25(OH)D levels were tested in separate laboratories individually prior to the performance test. The main method used was the protein binding immunoassay using “Elecsys^®^ Vitamin D total II” (Roche Diagnostics AG, Rotkreuz, Schweiz), which has been shown to accurately measure 25(OH)D levels [44]. Patients who had had their blood taken over three months prior to their performance test were excluded. All patients who had performed their performance test on a cycle ergometer were excluded. To ensure valid data, only athletes following the protocol mentioned above were included in this study. A total of 581 healthy athletes (284 females and 297 males) were included (Table 1 and Table 2). They were considered healthy as long as there were no medical contraindications for stress tests such as unstable cardiac disease or acute inflammatory disease [45,46]. Out of these only 224 female and 253 male athletes had their body fat measured. Body fat was therefore not included in further statistical analyses. Apart from body fat levels the data of all the athletes were complete.

Several statistical tests were performed to test whether vitamin D levels are associated with P_max_ or P_submax_. Males and females were analysed separately because the expected high variance in performance parameters would distort the results for possible correlations with 25(OH)D.

For further statistical analysis calculated BMI levels were divided into two groups of normal (BMI < 25 kg/m^2^) and overweight/obese (BMI ≥ 25 kg/m^2^), and a cut off value of 25(OH)D levels of 20 ng/mL [35], was used to separate athletes according to their vitamin D levels into groups with sufficient and “insufficient” vitamin D status.

A two-tailed statistical significance was accepted at the *p* < 0.05 level. Seasonal differences in 25(OH)D levels and P_max_ or P_submax_ were calculated using an ANOVA. Unpaired *t*-tests were used to assess the association of P_max_ and P_submax_ between groups of sufficient (serum 25(OH)D > 20 ng/mL) and “insufficient” (serum 25(OH)D ≤ 20 ng/mL) vitamin D status and to determine differences in P_max_, P_submax_ and serum 25(OH)D levels between athletes with normal BMI and overweight athletes as well as differences in P_max_ and P_submax_ between smokers and non-smokers. Additionally, an ANCOVA was performed to test for influencing variables (age, BMI, WTH and smoking status). Simple associations between primary, secondary and exploratory parameters were tested by Pearson correlations. Finally, stepwise multiple regression analyses were performed to determine whether the obtained exploratory parameters affected the correlation between vitamin D levels and performance. Four models were created for P_max_ and P_submax_ in male and female athletes, respectively. After performing regression analysis for 25(OH)D as an independent and P_max_ and P_submax_ as dependent variables, consecutively, the following parameter were added to the model: age, weekly training hours, BMI and nicotine abuse.

There were no outliers in the data as assessed by inspection of boxplots. Most of the parameters were normally distributed as verified by the Kolmogorov-Smirnov test and inspection of histograms. For those parameters that did not show normal distribution additionally non-parametric tests were performed for comparison, with similar results. All analyses were performed with IBM © SPSS software (Version 23, IBM Inc., Armonk, NY, USA).

## 3. Results

**Figure 1:**
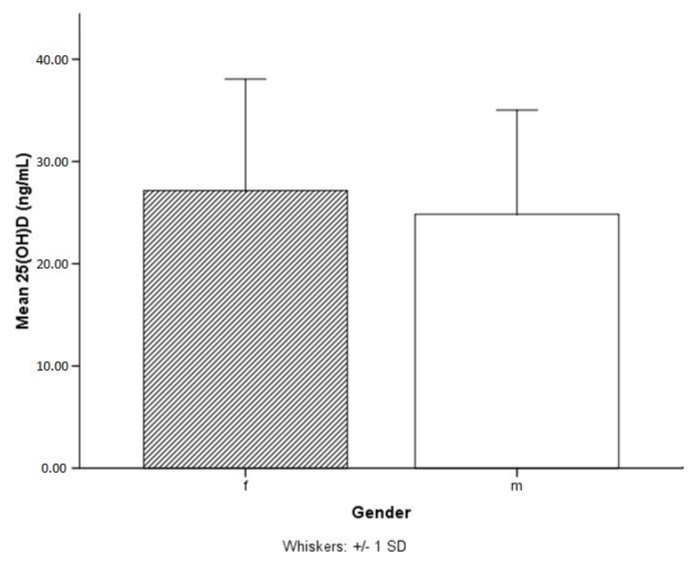
Mean serum 25(OH)D (25-hydroxyvitamin D) levels in females (f) with 27.17 ng/mL were found to be non-significantly higher than mean 25(OH)D levels in males (m) with 24.80 ng/mL.

Female athletes showed non-significantly higher serum 25(OH)D levels than male athletes (Figure 1). 75 females and 96 males had 25(OH)D levels ≤20 ng/mL. Therefore, according to the cut off levels defined by the IOM out of all the included athletes 26% of females and 32% of males would have an “insufficient” vitamin D status. However, if our data were analysed according to the US Endocrinology Society guidelines 66% of females and 76% of males would have insufficient vitamin D levels (≤30 ng/mL).

**Figure 2:**
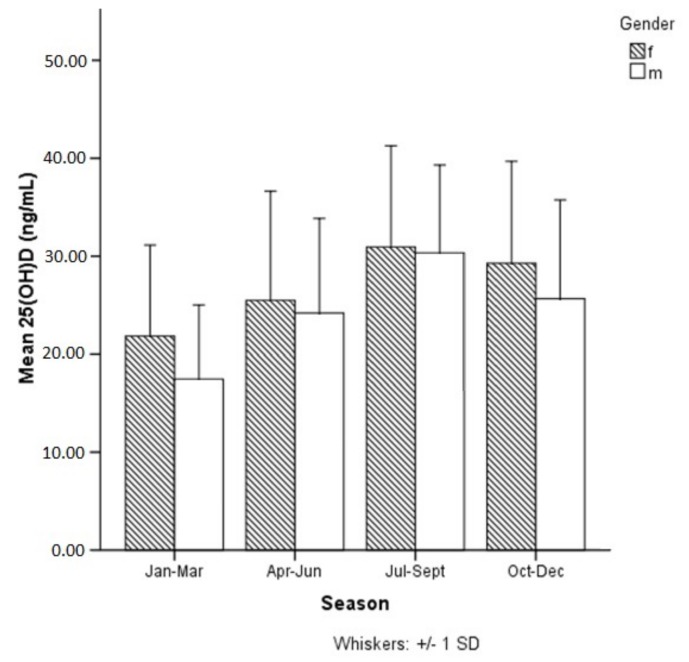
Significant variations in 25(OH)D (25-hydroxyvitamin D) levels between seasons (*p* = 0.000).

As expected, our results showed that 25(OH)D levels varied significantly between seasons with lowest values in winter and highest values in summer in female (F = 11.538, *p* = 0.000) and male athletes (F = 22.345, *p* = 0.000), respectively (Figure 2). Furthermore, our results showed that 57% percent of our athletes had vitamin D levels ≤30 ng/mL even during the summer months of July, August and September, however only 8% had levels ≤20 ng/mL.

**Figure 3:**
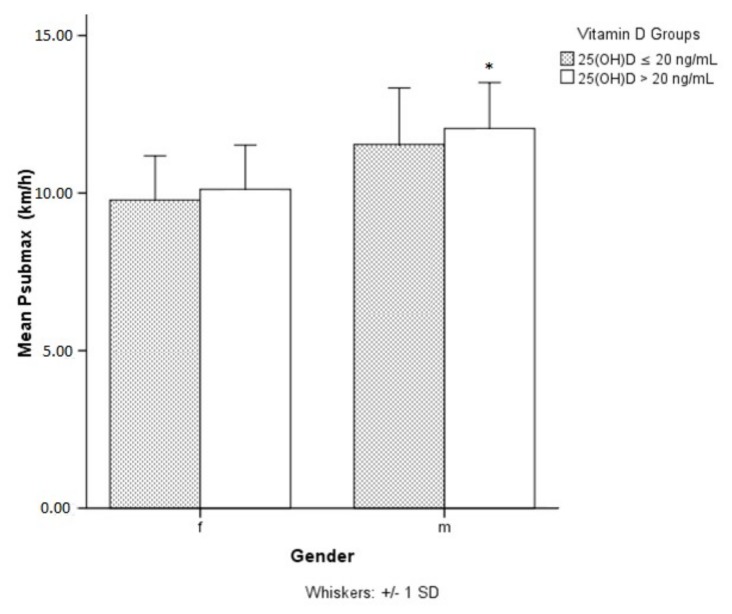
Significant differences (*p* = 0.045) in submaximal performance between groups of sufficient vitamin D status (25(OH)D > 20 ng/mL) and insufficient vitamin D status (25(OH)D ≤ 20 ng/mL) in males but not in females. 25(OH)D: 25-hydroxyvitamin D; * submaximal performance significantly higher in male athletes with 25(OH)D levels >20 ng/mL.

There were no seasonal differences for P_max_ and P_submax_ (data not shown) but male athletes with sufficient (>20ng/mL) 25(OH)D levels showed significantly higher P_submax_ levels compared to those with levels ≤ 20 ng/mL (Figure 3). In females the difference in P_submax_ was insignificant (*p* = 0.08). Any detected differences between vitamin D groups (≤20 ng/mL vs. > 20 ng/mL) in P_max_ and P_submax_ for both male and female athletes were insignificant when controlled for modifying variables (age, BMI, WTH, smoking status) (data not shown).

Concordant with other findings [47] P_max_ and P_submax_ were significantly lower in overweight males compared to those with normal BMI (*p* < 0.001) with no significant difference in 25(OH)D levels between the two groups (data not presented). In female athletes we found significant differences in P_max_, P_submax_ and 25(OH)D (Table 3). 

No significant differences in P_max_ and P_submax_ were found between smokers and non-smokers in male and female athletes (data not shown).

In female athletes significant correlations at the *p* = 0.05 level were found for 25(OH)D and P_max_ (r = 0.143) as well as for 25(OH)D and P_submax_ (r = 0.141), while in male athletes only the correlation between 25(OH)D and P_submax_ (r = 0.139) reached the level of significance. Even though significant correlations were shown between 25(OH)D and P_max_ and P_submax_ in female athletes and between 25(OH)D and P_max_ in male athletes, correlation coefficients were low. Furthermore, after applying a Bonferroni correction for multiple testing with a new p value of < 0.001 only few correlations would remain significant (Table 4 and Table 5).

In multiple regression analyses different models were used to study the effect of WTH, BMI and smoking status on the association between 25(OH)D and performance (Table 6 and Table 7). Unexpectedly, the results differed greatly between males and females. In male athletes the correlation between 25(OH)D and P_max_ became more significant in model 2 once age was added as an independent parameter (*p* = 0.006) (Table 7). Further inclusion of other covariates slightly increased the significance. The correlation between 25(OH)D and P_submax_ was already significant in model 1 (*p* = 0.017) and remained significant. In female athletes the significant correlation between 25(OH)D and P_max_ disappeared after adding WTH to the analysis (Table 6). After adding BMI and nicotine abuse in model 4 the correlation becomes even more insignificant (*p* = 0.923). Multiple regression analysis for P_submax_ and vitamin D in female athletes revealed similar results.

## 4. Discussion

The major findings of our study are that there is a small but significant correlation between vitamin D status and parameters of endurance performance, especially in male athletes. In female athletes the correlation seems to be influenced to a greater extent by other biological and lifestyle factors like WTH, BMI and nicotine abuse. Accordingly, we found significant correlations between 25(OH)D and WTH as well as BMI in females only. Compared to earlier studies that have yielded controversial results as to whether vitamin D deficiency is linked to physical performance in athletes our findings show only a mild significant correlation between 25(OH)D and performance parameters. Most studies have shown a correlation between vitamin D status and muscle strength parameters [22,23,24,25,48], it is therefore possible that vitamin D has a greater effect on muscle performance than it has on endurance parameters. 

A systematic review and meta-analysis from 2014 including 23 articles with 2313 athletes from the UK, Ireland, Spain, France, the USA, Australia and Middle Eastern Countries showed a prevalence rate of 56% for vitamin D inadequacy (serum 25(OH)D levels <80 nmol/L = 32 ng/mL) [34]. Compared with these results, our findings revealed even higher prevalence rates. Furthermore, when dividing our data into groups of athletes with sufficient and insufficient vitamin D status, the choice of cut-off levels markedly influences the prevalence of athletes with vitamin D deficiency. The percentages of athletes with sufficient and insufficient vitamin D status are almost exactly reversed when cut-off levels are changed from 20 to 30 ng/mL

The issue of optimal vitamin D cut-off levels has been intensively discussed over the last decades and remains unsolved. For example, based on biochemical findings Heaney at el. suggested that optimal serum concentrations start at the turning point in 25(OH)D kinetics of 88 nmol/L (35.2 ng/mL) [49]. In terms of bone mineral density (BMD), lower-extremity function, falls, dental health and colorectal cancer prevention Bischoff-Ferrari et al. [3] found 25(OH)D concentrations to be most advantageous starting from 75 nmol/L with best concentrations between 90 and 100 nmol/L (36–40 ng/mL), hence also supporting the claim of a higher cut off level. 

The German Nutrition Society [50] proposes a daily vitamin D intake of 20 μg for adolescents and adults under 65 years and 20–25 μg per day for populations older than 65 years in case of absent endogenous vitamin D synthesis as the majority of people were found to achieve 25(OH)D levels >50 nmol/L with a supplementary intake of 20 μg/d during the winter months in a study published in 2008 by Cashman et al. [51].

In our data an expected variance in vitamin D levels was observed throughout the seasons, but seasonal differences were detected in neither P_max_ nor P_submax_. These findings stand partly contradictory to earlier findings that have detected significant differences in physical fitness parameter dependent on season or exposure to UVB radiation [52,53,54,55,56,57]. While our data include a sample of hobby athletes most of the cited studies have included a small number of elite athletes or have analysed other outcome parameter for physical performance (i.e., VO_2_max, heart rate variability, strength). Direct comparison with these findings might therefore not be suitable to interpret our results.

In accordance with this, it is not surprising that we found no significant correlation between 25(OH)D and P_max_ for both male and female athletes. However, the findings for P_submax_ revealed a small correlation that was significant in men and almost significant in women, suggesting that P_submax_ and vitamin D levels might be linked in some way.

An interesting result was the significant correlation between 25(OH)D and WTH in female but not male athletes. One plausible explanation for this discrepancy could be that female athletes do more outdoor training. However, we did not find studies showing these gender specific differences in training habits. An earlier study by Sparling and Cureton revealed no significant differences in the effect of performance determining variables, like cardiorespiratory capacity, body fat and running economy on distance running performance between similarly trained males and females. The authors however found that body fat accounted for the greatest part of gender differences in running performance [58].

As opposed to male athletes, the data for female athletes also showed a slight significant negative correlation between 25(OH)D levels and BMI. There is a known difference in distribution of body mass between men and women. While the percentage of lean body mass in men is significantly higher, women have an average of 7–9% higher amount of body fat [59]. A higher BMI in female athletes is therefore more likely to be associated with higher body fat. As vitamin D is mostly stored in adipose tissue, this might be a possible explanation for the negative correlation with BMI in female athletes [60]. Also, inhibition of adipogenesis by 1,25(OH)_2_D has been discussed [61]. VDR and hydroxylation enzymes have also been detected in adipose tissue indicating a possible regulative role of vitamin D [11]. Whether vitamin D levels are a consequence of higher body fat or might possibly contribute to it is not clear.

The most prominent gender differences were shown in multiple regression analyses. In male athletes, for both maximal and submaximal performance, age, weekly training hours and BMI seem to stabilize the correlation with 25(OH)D. By contrast, the significant correlation between 25(OH)D and P_submax_ or 25(OH)D and P_max_ in female athletes is hardly influenced by age but is reduced remarkably by weekly training hours and BMI. These findings match the correlations mentioned above. Seemingly, weekly training hours and BMI in female athletes affect 25(OH)D levels in a way that decreases their effect on performance. Although a specific reason for this effect remains unclear, different factors might have played a role like more relevant body weight changes in women compared to men or differences in health awareness that lead to higher supplementation of vitamin D. Considering that elite athletes in particular have been found to rely on supplementation to reach their required intake for several minerals and vitamins [62] it cannot be ruled out that athletes who do more training take more supplementation and might therefore show a better performance. However, given the cross-sectional study design it is not possible to evaluate causal relationships in this triangle. Nevertheless, the primary aim of this study was to detect correlations between vitamin D levels and performance.

Other gender specific factors that should be considered to influence the relationship between vitamin D and performance include biochemical factors, like hormonal differences. For example, Heaney et al. reported that vitamin D binding protein (DBP) levels are increased during pregnancy and estrogen therapy [49]. However, due to the tight self-regulation through feedback mechanisms the concentration of free 1,25(OH)_2_D does not change. Intracellular vitamin D binding proteins (IDBPs) on the other hand directly modify intracellular vitamin D pathways by mediating the transport of 25(OH)D to mitochondrial 1α-hydroxylase and are also known to bind estradiol. If higher estradiol levels lead to competitive replacement of 25(OH)D at the binding site, this could result in gender differences in vitamin D metabolism. Further studies would be needed to investigate this on a molecular level.

Similarly, two studies have found gender related differences in FGF-23 in infants [63,64]. Considering the newest finding on the role of FGF-23 in vitamin D regulation [14], the gender differences found might be partly due to this connection. However another study group identified significant associations between FGF-23 and age but not gender [65]. Recent genomic investigations by Miettinen et al. [66] showed that age is involved in the association between SPNs and 25(OH)D, however no significant differences for gender were detected.

The main limitation in our study is that no interpretation on causality is possible, as is the case in all observational studies. In terms of data it also must be stated that the included measurements of serum 25(OH)D levels have been made at several individual laboratories and therefore no standardized measurement method could be ensured. However, all measurements have been undertaken in qualified and listed laboratories in Vienna, thus guaranteeing certified quality.

## 5. Conclusions

To conclude, the effects of vitamin D on muscle and physical performance are a topic still under investigation. While numerous studies have concentrated on specific targets of vitamin D metabolites and their intracellular effects, the definite consequences of those effects remain unclear in many cases. Observational studies that have been conducted have shown that vitamin D status might correlate with several aspects of physical performance but need to be followed by randomized controlled trials with sufficient sample size, predetermined cut-off levels and precise research aims. Moreover, as 25(OH)D cut-off levels determine the necessity to prescribe vitamin D supplementation in daily practice, a consensus in this field is sorely needed. Future studies might aim directly at answering the question whether vitamin D levels of 20 or 30 ng/mL make a difference in performance as well as many other health outcome parameters.

## Figures and Tables

**Table 1:** Descriptive statistics of all female athletes.

	N	Minimum	Maximum	Mean	SD	Variance
Age	284	18	65	38.66	9.82	96.51
Bodyweight (kg)	284	45.1	99.4	63.42	9.12	83.24
Height (cm)	284	152	188	166.91	6.13	37.62
BMI (kg/m^2^)	284	17.5	34.2	22.76	2.84	8.09
25(OH)D (ng/mL)	284	7.5	66.5	27.17	10.89	118.57
Weekly training (h)	284	0	15	5.20	1.53	2.34
P_max_ (km/h)	284	6.7	18.7	12.94	1.96	3.83
P_submax_ (km/h)	284	5.3	16.2	10.02	1.40	1.95

BMI: body mass index; 25(OH)D: 25-hydroxyvitamin D; maximal (P_max_) and submaximal physical performance (P_submax_).

**Table 2:** Descriptive statistics of all male athletes.

	N	Minimum	Maximum	Mean	SD	Variance
Age	297	18	64	40.54	9.16	83.95
Bodyweight (kg)	297	47.6	149.4	80.12	11.43	130.71
Height (cm)	297	155.0	198.0	180.40	6.71	45.04
BMI (kg/m^2^)	297	18	41.3	24.53	2.81	7.94
25(OH)D (ng/mL)	297	5.2	64.5	24.80	10.15	103.09
Weekly training (h)	297	2	20	6.21	2.21	4.89
P_max_ (km/h)	297	8.7	22.0	15.81	1.98	3.92
P_submax_ (km/h)	297	7.1	17.0	11.85	1.59	2.523

BMI: body mass index; 25(OH)D: 25-hydroxyvitamin D; maximal (P_max_) and submaximal physical performance (P_submax_).

**Table 3:** Differences in P_max_, P_submax_ and 25(OH)D levels between female athletes with normal and high BMI.

	F	*p*-Value	BMI (kg/m^2^)	N	Mean ± SD
P_max_ (km/h)	5.075	0.000	<25 (normal)	234	13.23 ± 1.78
			≥25 (overweight)	50	11.57 ± 2.19
P_submax_ (km/h)	2.384	0.000	<25 (normal)	234	10.2 ± 1.32
			≥25 (overweight)	50	9.21 ± 1.49
25(OH)D (ng/mL)	0.445	0.012	< 25 (normal)	234	27.91 ± 10.68
			≥25 (overweight)	50	23.67 ± 11.29

*t*-test for BMI (normal and overweight) groups of normal weight (<25) and overweight (≥25) as independent variables; 25(OH)D: 25-hydroxyvitamin D.

**Table 4:** Pearson correlations of included variables in female athletes.

		VitD Groups	25(OH)D (ng/mL)	P_max_ (km/h)	P_submax_ (km/h)	Age	WTH (h)	BMI	Nicotine
VitD groups	Pearson’s r	1	**0.680**	0.087	0.104	−0.054	0.125	**−0.205**	−0.106
	*p*-value		0.000	0.143	0.080	0.364	0.036	0.000	0.075
25(OH)D (ng/mL)	Pearson’s r	**0.680**	1	0.143	0.141	−0.051	**0.230**	−0.149	−0.164
	*p*-value	0.000		0.016	0.017	0.390	0.000	0.012	0.006
P_max_ (km/h)	Pearson’s r	0.087	0.143	1	**0.897**	**−0.391**	**0.400**	**−0.324**	−0.100
	*p*-value	0.143	0.016		0.000	0.000	0.000	0.000	0.092
P_submax_ (km/h)	Pearson’s r	0.104	0.141	**0.897**	1	**−0.260**	**0.439**	**−0.270**	−0.135
	*p*-value	0.080	0.017	0.000		0.000	0.000	0.000	0.023
Age	Pearson’s r	−0.054	−0.051	**−0.391**	**−0.260**	1	−0.160	0.175	0.006
	*p*-value	0.364	0.390	0.000	0.000		0.007	0.003	0.920
WTH (h)	Pearson’s r	0.125	**0.230**	**0.400**	**0.439**	−0.160	1	−0.132	−0.014
	*p*-value	0.036	0.000	0.000	0.000	0.007		0.026	0.809
BMI	Pearson’s r	**−0.205**	−0.149	**−0.324**	**−0.270**	0.175	−0.132	1	−0.008
	*p*-value	0.000	0.012	0.000	0.000	0.003	0.026		0.887
Nicotine	Pearson’s r	−0.106	−0.164	−0.100	−0.135	0.006	−0.014	−0.008	1
	*p*-value	0.075	0.006	0.092	0.023	0.920	0.809	0.887	

VitD groups (≤20 ng/mL, >20 ng/mL); 25(OH)D: 25-hydroxyvitamin D; WTH: weekly training hours; BMI (normal and overweight); Nicotine (abuse yes or no). All findings significant at the < 0.001 level are bold.

**Table 5:** Pearson correlations of included variables in male athletes.

		VitD Groups	25(OH)D (ng/mL)	P_max_ (km/h)	P_submax_ (km/h)	Age	WTH (h)	BMI	Nicotine
**VitD groups**	Pearson’s r	1	**0.686**	0.077	0.125	0.071	0.093	−0.087	−0.026
	*p*-value		0.000	0.184	0.031	0.220	0.109	0.134	0.654
**25(OH)D (ng/mL)**	Pearson’s r	**0.686**	1	0.102	0.139	0.116	−0.013	−0.029	0.000
	*p*-value	0.000		0.080	0.017	0.045	0.817	0.619	0.996
**P_max_ (km/h)**	Pearson’s r	0.077	0.102	1	**0.851**	**−0.378**	**0.401**	**−0.355**	−0.065
	*p*-value	0.184	0.080		0.000	0.000	0.000	0.000	0.267
**P_submax_ (km/h)**	Pearson’s r	0.125	0.139	**0.851**	1	−0.168	**0.452**	**−0.316**	−0.086
	*p*-value	0.031	0.017	0.000		0.004	0.000	0.000	0.137
**Age**	Pearson’s r	0.071	0.116	**−0.378**	−0.168	1	−0.113	0.087	−0.135
	*p*-value	0.220	0.045	0.000	0.004		0.052	0.135	0.020
**WTH (h)**	Pearson’s r	0.093	−0.013	**0.401**	**0.452**	−0.113	1	−0.146	−0.067
	*p*-value	0.109	0.817	0.000	0.000	0.052		0.012	0.252
**BMI**	Pearson’s r	−0.087	−0.029	**−0.355**	**−0.316**	0.087	−0.146	1	0.105
	*p*-value	0.134	0.619	0.000	0.000	0.135	0.012		0.070
**Nicotine**	Pearson’s r	−0.026	0.000	−0.065	−0.086	−0.135	−0.067	0.105	1
	*p*-value	0.654	0.996	0.267	0.137	0.020	0.252	0.070	

VitD groups (≤20 ng/mL, >20 ng/mL); 25(OH)D: 25-hydroxyvitamin D; WTH: weekly training hours; BMI (normal and overweight); Nicotine (abuse yes or no). All findings significant at the < 0.001 level are bold.

**Table 6:** Associations between 25(OH)D levels and P_max_/P_submax_ in female athletes.

	β		*p*-Value	
	P_max_	P_submax_	P_max_	P_submax_
**Model 1** (unadjusted)				
25(OH)D	0.143	0.141	0.016	0.017
**Model 2** (adjusted for age)				
25(OH)D	0.124	0.128	0.024	0.026
**Model 3** (adjusted for age + WTH)				
25(OH)D	0.049	0.039	0.349	0.467
**Model 4** (adjusted for age + WTH + BMI + nicotine abuse)				
25(OH)D	0.005	−0.007	0.923	0.903

**Table 7:** Associations between 25(OH)D levels and P_max_/P_submax_ in male athletes.

	β		*p*-Value	
	P_max_	P_submax_	P_max_	P_submax_
**Model 1** (unadjusted)				
25(OH)D	0.102	0.139	0.080	0.017
**Model 2** (adjusted for age)				
25(OH)D	0.148	0.160	0.006	0.005
**Model 3** (adjusted for age + WTH)				
25(OH)D	0.148	0.161	0.003	0.002
**Model 4** (adjusted for age + WTH + BMI + nicotine abuse)				
25(OH)D	0.138	0.152	0.003	0.002
